# Preparing for cascading hazards in High Mountain Asia

**DOI:** 10.1093/nsr/nwaf523

**Published:** 2025-11-20

**Authors:** Xin Wang, Xuanmei Fan, Kushanav Bhuyan

**Affiliations:** State Key Laboratory of Geohazard Prevention and Geoenvironment Protection, Chengdu University of Technology, China; State Key Laboratory of Geohazard Prevention and Geoenvironment Protection, Chengdu University of Technology, China; State Key Laboratory of Geohazard Prevention and Geoenvironment Protection, Chengdu University of Technology, China

## Abstract

High Mountain Asia stands out as the global epicentre of cryospheric risk, and is possible to provide a model for global resilience in a rapidly warming world. Typesetting of author information: set at the end of the article. use the same type as that of the Perspective articles.

In 2021, a rock-ice wedge collapsed from Ronti Peak above Chamoli in India, vaporizing glacial ice and generating a debris flood that killed more than 200 people and destroyed two hydropower projects [1]. Nepal’s 2013 Seti Khola disaster showed similar dynamics, as an ice-rock avalanche transformed into a devastating flood within minutes [2]. In the Sedongpu Gully in China's Xizang Autonomous Region, at least five major cascading slope failures between 2017 and 2023 repeatedly dammed and breached the Yarlung Tsangpo River, producing surges that reshaped downstream valleys [3]. The 2023 Sikkim glacial lake outburst flood (GLOF) propagated >300 km down the Teesta River basin [4], destroying the Chungthang dam, while the 2025 Gyirong GLOF devastated a critical border port between China and Nepal [5] (Fig. 1).

**Figure 1.**
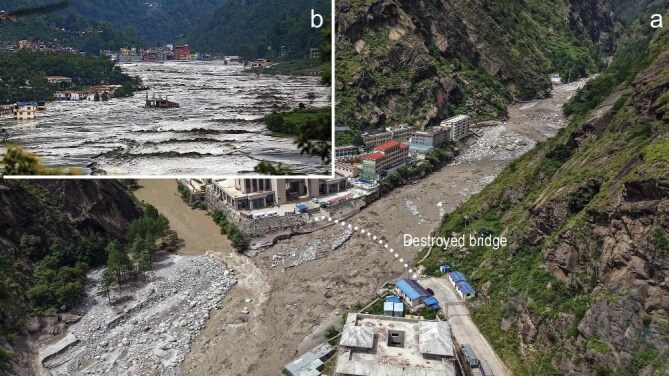
GLOF on 8 July 2025 resulting from a supraglacial lake system developed atop the Purepu Glacier in Xizang, China. (a) The Sino-Nepal Friendship Bridge at Gyirong Port was swept away by the GLOF. (b) Floodwaters inundated downstream villages in Nepal. Image credits: (a) Prakash Chandra Timilsena; (b) Agence France-Presse.

Together, these catastrophes show that cryospheric instabilities across High Mountain Asia rarely remain isolated: they unfold as dynamic, multistage chains with one process triggering another and their downstream impacts now extend far beyond historical experience. They mark the emergence of a new hazard regime in the world’s cold-mountain regions, demanding that cascading processes be recognized as systemic risks rather than exceptional anomalies.

## ESCALATING DRIVERS AND SYSTEMIC RISKS IN HIGH MOUNTAIN ASIA

High Mountain Asia stands out as the global epicentre of cryospheric risk. Nowhere else do retreating glaciers, degrading permafrost, changing meltwater patterns and extreme rainfall converge with such dense populations, rapidly expanding infrastructure and fragile governance systems.

Compared with the European Alps, where institutional and financial capacity enables more robust responses, High Mountain Asia faces cascading hazards with weaker monitoring, fragmented authority and limited resources. This asymmetry highlights both the urgency and the global responsibility: cascading hazards in High Mountain Asia are not a regional concern, but a planetary risk node in a warming climate.

**Figure 2.**
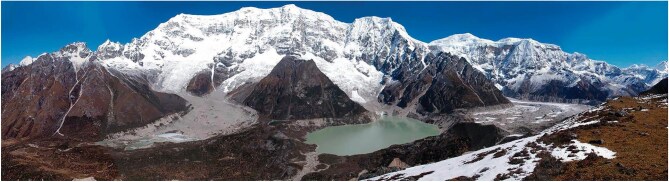
Lunana region in the Bhutan Himalaya, home to the country’s most hazardous glacial lakes, is highly sensitive to climate warming as rapidly melting glaciers raise lake levels and heighten the risk of outburst floods. Image credit: Koji Fujita.

Climate change is accelerating both the frequency and complexity of these hazard chains (Fig. 2). The compounding of cryospheric and geomorphic instabilities produces non-linear sequences of events, the consequences of which extend far beyond historical baselines [6]. Expanding hydropower and urban development in high-mountain valleys can further amplify rather than mitigate these risks [7], transforming single events into multistage cascades that exceed the design expectations of infrastructure. To persist with single-hazard monitoring and response frameworks is not only inadequate, but a deliberate neglect of the lessons written by past tragedies.

## EARLY DETECTION

A transformation of early warning is urgently needed. Hazard-based alerts must give way to impact-based, scenario-driven forecasting. Monitoring networks that combine satellite remote sensing with the Global Navigation Satellite System (GNSS), radar, seismic and thermal sensors should feed into models capable of simulating credible process chains.

Decision-makers require not vague statements about instability, but concrete forecasts of when, where and how cascading impacts will unfold. Without scenario portfolios that translate science into decision-ready forecasts, early-warning systems remain symbolic rather than life-saving.

The 2025 Blatten event in the Swiss Alps illustrates what is possible when science, technology and governance align. A 2-week sequence of rockfalls added millions of cubic metres of debris onto the Birchgletscher, triggering the collapse of ∼3 million cubic metres of glacier ice; together, nearly 10 million cubic metres of rock-ice material buried most of the village and dammed the Lonza River [8]. Yet, because authorities acted on alarming signals detected by GNSS, InSAR, seismic and camera networks, the entire population was evacuated and no fatalities occurred, which is a rare success in cryospheric hazard mitigation.

This experience underscores the value of real-time data integration, predefined action thresholds and trusted communication channels, all of which remain limited across High Mountain Asia.

## COORDINATED ACTION

Policy frameworks must evolve with the science. Agility must replace rigidity: disaster protocols should adapt as understanding advances, local authority should be empowered to act quickly and financing mechanisms must be flexible enough to support the rapid deployment of pilot systems.

Cascading hazards cut across water, energy, transport, environment and emergency management, and only interoperable data platforms and coordinated decision rules can prevent institutional fragmentation from amplifying disaster impacts. Existing global frameworks acknowledge the need for multi-hazard approaches, yet few explicitly address cascading interactions. Recognizing and operationalizing these linkages are crucial to strengthen both policy coherence and practical capacity. For example, cascade-aware risk appraisal should be mandatory in environmental assessments for hydropower and transport projects in high mountains; without it, infrastructure risks becoming an amplifier of disaster rather than a symbol of resilience.

Cascading hazards are simultaneously geophysical, social and political. Geoscientists, engineers, social scientists, emergency planners and financiers must jointly design monitoring protocols, response thresholds and training programmes. Expertise in hazard cascades should be institutionalized in current education and professional training, ensuring that knowledge does not vanish between research and practice.

The contrast between Chamoli, where early signals went unheeded, and Blatten, where thresholds and evacuations worked, underscores a simple truth: science saves lives only when coupled with legitimate authority and public trust.

Because many of these hazards cascade in High Mountain Asia across national borders, regional cooperation is also essential. Shared monitoring stations, real-time data exchange and joint emergency protocols between neighbouring countries are prerequisites for managing transboundary risk.

A chronic bottleneck for cascading hazard response is financing. We propose the establishment of demonstration basins—internationally supported observatories across High Mountain Asia that integrate monitoring, modelling and community engagement. These basins would serve as laboratories for scalable solutions, with transparent multi-stakeholder financing. Too often, promising pilots in Nepal and elsewhere collapse due to fragmented donor support and short-term funding. Without structural reforms and sustained investment, cascading hazard preparedness will remain patchwork and temporary rather than systemic.

## TOWARD SYSTEMIC RESILIENCE IN A WARMING MOUNTAIN WORLD

**Figure 3.**
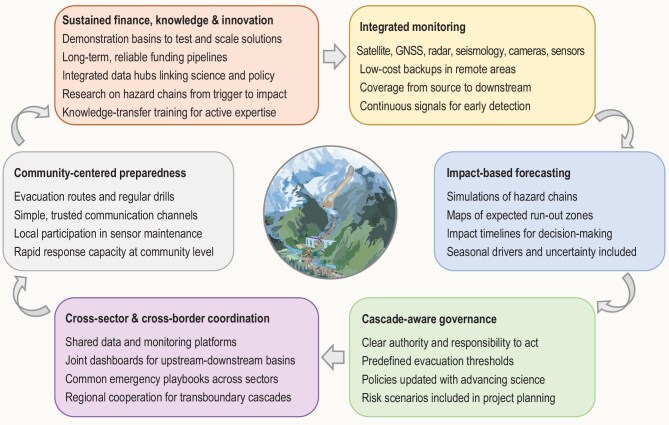
Preparedness for cascading hazards in High Mountain Asia.

Cascading hazards must therefore be recognized as a distinct category of systemic risk within global climate adaptation and disaster governance (Fig. 3). High Mountain Asia represents the frontline of this transformation, where the collision of cryospheric change, infrastructure expansion and fragile governance makes systemic risk visible in real time. They are no longer rare outliers, but the defining disasters of the high-mountain cryosphere in the twenty-first century.

The scientific and technological tools to detect, model and forecast them already exist, as do lessons from both catastrophic failures and successful interventions. What is missing are political will, institutional agility and cross-border solidarity.

If High Mountain Asia can pioneer cascade-aware monitoring, forecasting and financing frameworks, then it will not only safeguard its own populations, but also provide a model for global resilience in a rapidly warming world. If, however, hazards continue to be framed as isolated and static, then Chamoli-like tragedies will be repeated.
